# Characterization of Hexagonal Close-Packed Zn-Cu-Ti Alloy Pyramid Drawpieces in Single-Point Incremental Sheet Forming Process

**DOI:** 10.3390/ma18133078

**Published:** 2025-06-28

**Authors:** Łukasz Kuczek, Krzysztof Żaba, Tomasz Trzepieciński, Maciej Balcerzak, Vít Novák

**Affiliations:** 1 Department of Metal Working and Physical Metallurgy of Non-Ferrous Metals, Faculty of Non-Ferrous Metals, AGH University of Krakow, al. Adama Mickiewicza 30, 30-059 Krakow, Poland; krzyzaba@agh.edu.pl (K.Ż.); balcerzak@agh.edu.pl (M.B.); 2 Department of Manufacturing Processes and Production Engineering, Faculty of Mechanical Engineering and Aeronautics, Rzeszow University of Technology, al. Powst. Warszawy 8, 35-959 Rzeszów, Poland; 3 Department of Manufacturing Technology, Faculty of Mechanical Engineering, Czech Technical University in Prague, Technická 4, 166 07 Prague, Czech Republic; vit.novak@fs.cvut.cz

**Keywords:** formability, forming limit angle, incremental sheet forming, SPIF, surface roughness, Zn-Cu-Ti alloy

## Abstract

Incremental sheet forming technology is finding increasing application in the production of components in many industries. This article presents the analysis of the formability of 0.68-mm-thick Zn-Cu-Ti alloy sheets during the single-point incremental forming (SPIF) of pyramid-shaped drawpieces. Basic mechanical properties of sheets were determined in a uniaxial tensile test. Formability tests were carried out using the Erichsen and Fukui methods. SPIF tests were carried out under the conditions of variable process parameters: tool diameter (12 and 20 mm), feed rate (500–3000 mm/min), tool rotational speed (250–3000 rpm), and step size (0.1–1.2 mm). The effect of SPIF process parameters on the value of basic mechanical parameters, maximum deviation of the measured wall profile from the ideal profile, limit-forming angle, and surface roughness of pyramid-shaped drawpieces was determined. It was found that increasing the step size resulted in a decrease in the value of the limit-forming angle. Both the step size and the tool rotational speed contribute to the increase of the maximum wall deviation. However, the use of higher feed rates and a larger tool diameter caused its reduction. Higher values of arithmetic mean surface roughness Ra were found for the outer surface of drawpieces. The use of a smaller step size with a larger tool diameter caused a reduction in the Ra value of the drawpiece wall. Based on the obtained results, it can be concluded that the Zn-Cu-Ti alloy demonstrates good suitability for SPIF when proper process parameters and sheet orientation are selected. An appropriate combination of tool diameter, feed rate, step size, and sample orientation can ensure the desired balance between dimensional accuracy, mechanical strength, and surface quality of the formed components.

## 1. Introduction

Incremental sheet forming (ISF) has gained much interest in industrial applications and an increase in the number of publications devoted to the analysis of this process in the last 10 years. Depending on the kinematics of the forming process, ISF can be generally divided into single-point incremental forming (SPIF) and two-point incremental forming (TPIF) [1,2]. The advantages of ISF include high geometric flexibility, high formability due to localized deformation, and no need for matched dies. ISF uses a simple hemispherical or ball-ended tool. With low cost for prototyping and small batches, the ISF technique is ideal for custom or low-volume production [3]. In warm forming variants, the ISF uses localized heating of the workpiece to improve formability [4]. Similar to any forming technology, ISF, in addition to its advantages, also has disadvantages such as limited wall thickness control, limited dimensional accuracy, works best with ductile materials, and needs a CNC-controlled setup for precision and repeatability [5,6]. Surface quality of ISF-ed components depends on tool path and initial surface roughness of the workpiece [7,8].

Both ductile materials and difficult-to-deform materials such as titanium alloy, magnesium and its alloys, superalloys and zinc alloys can be subjected to incremental sheet forming [9]. The main disadvantage of Hexagonal Close-Packed (HCP) difficult-to-deform materials is their low formability at ambient temperature, which results from the type of crystallographic lattice [10]. Poor ductility of HCP alloy in cold forming is attributed to the lack of adequate independent slip systems to accommodate plastic deformation and their complex dislocation structures [11]. Cold forming of HCP alloys is a challenge in SPIF/TPIF processes and is associated with the appropriate selection of the process parameter values. The rolling process of HCP alloy causes strong anisotropy in the material structure [12,13]. In relation to the Zn-Cu-Ti alloy sheet studied in this work, the effect of material texture on formability was studied in [14].

The selection of SPIF process parameters and lubrication conditions is necessary to ensure the appropriate formability of the sheet metal and to obtain the drawpieces with the specified dimensional and shape quality [15]. The basic parameters of the incremental forming process include tool rotational speed, feed rate, tool diameter, tool path, and step size [16,17,18]. The selection of friction conditions consists in selecting a lubricant adapted to the formed sheet metal and its roughness as well as the processing temperature [19,20]. Generally, similar lubricants are used in ISF as in conventional sheet metal forming (SMF) processes [21]. In recent years, many works have been published on the selection of optimal parameters of the ISF processes. The main methods used for this purpose are analysis of variance [22], the Taguchi method [23], response surface methodology [24], and artificial intelligence methods (machine learning [25], artificial neural networks [26], genetic algorithms [27], etc.). Szpunar et al. [28] investigated the influence of forming process parameters on the surface roughness (Sa, Sz) of the outer surface of Ti-6Al-4V titanium alloy sheets, which consist of a majority of primary *HCP*. It was found that at low tool feed rates, reducing the step size caused an increase in the Sz parameter. At high tool feed rates, this effect was negligible. In another article, Szpunar et al. [29] studied the effect of process parameters on the friction coefficient when warm SPIF process of Ti-6Al-4V titanium alloy sheets. It was concluded that adjusting the feed rate can help to optimize the frictional conditions of the SPIF process. Zhang et al. [30] analyzed SPIF of the HCP-structured AZ31 magnesium alloy sheets at elevated temperatures. They concluded that cross-rolling sheets are much more suitable for the warm SPIF process. An et al. [31] developed a multistage warm SPIF process for AZ31 alloy sheets. They determined the optimal forming temperature (250 °C) and process parameters. It was found that formability is improved with the increases of tool diameter and forming temperature. Rai et al. [32] studied the geometrical accuracy in the SPIF of difficult-to-deform Inconel 625 superalloy sheets. The multi-stage approach allowed for increasing forming limits and controlling the thickness thinning of sheets. The poor workability of titanium grade 2 in cold SPIF is attributed to its HCP crystal structure. Araújo et al. [33] investigated the effect of SPIF process parameters on the failure limit when forming titanium grade 2 facial implants. They concluded that the interaction between fracture and instability is consistent with the concept of local necking, which was also confirmed by Embury et al. [34]. Kumar et al. [35] developed hot-assisted SPIF to form HCP AZ31B magnesium alloy sheets. They numerically and experimentally confirmed that the fracture depth increases as the temperature increases.

Despite extensive studies on the incremental forming of titanium, magnesium, and other difficult-to-deform HCP materials, there is a distinct lack of experimental investigations focused on Zn-Cu-Ti alloys in the SPIF process. The existing literature does not provide sufficient information on how process parameters influence formability, wall geometry accuracy, and surface quality in this specific alloy group. This knowledge gap motivated the present study, which aims to comprehensively evaluate the suitability of Zn-Cu-Ti alloy for cold SPIF and establish optimal forming parameters for achieving high-quality components.

Zn-Cu-Ti alloys are used in industrial applications that require structurally complex components such as precision connectors, decorative elements, and corrosion-resistant parts, where high formability and strength are needed. Due to their HCP structure and anisotropic properties resulting from rolling, these materials are typically difficult to form at room temperature using conventional methods [36,37]. However, the localized deformation mechanisms inherent in SPIF offer superior formability compared to traditional sheet forming techniques [38], which makes SPIF a promising approach for Zn-Cu-Ti alloys. This study investigates whether the advantages of SPIF can be effectively utilized to overcome the inherent limitations of Zn-Cu-Ti in cold forming conditions.

In this paper, an approach to cold SPIF of highly anisotropic HCP Zn-Cu-Ti alloy sheets is presented for the first time. The main objective of the research was to experimentally determine the effect of tool diameter, feed rate, tool rotational speed, and step size on the formability limit of sheet metals when forming the pyramid drawpieces. The main research tasks include the study of the influence of forming parameters on the mechanical properties of the drawpieces and their surface roughness, as well as the analysis of variance (ANOVA) of the relationship between the input and output parameters of the SPIF process.

## 2. Materials and Methods

### 2.1. Material

The material for the tests was a Zn-Cu-Ti zinc alloy sheet metal with the chemical composition given in Table 1. The 0.68-mm-thick sheet was supplied by MetZink (Poznań, Poland) in a soft state.

The mechanical properties of the sheet were determined in a static uniaxial tensile test at room temperature according to the recommendations of the EN ISO 6892-1 standard [39]. For this purpose, samples were cut with three orientations relative to the rolling direction (RD) of the sheet metal: 0°, 45° and 90°. The gauge width and gauge length were 12.5 mm and 50 mm, respectively. The length of the sample was 75 mm. The test was carried out with a constant strain rate of 5 × 10^−3^ s^−1^.

The plastic anisotropy of the sheet metals was also tested according to the recommendations of the EN ISO 10113 standard [40]. The tests were carried out under the same conditions and on samples of the same dimensions as in the uniaxial tensile test. The anisotropy coefficient r (r-value) for a given sample orientation relative to the RD, the coefficient of normal anisotropy $\bar{r}$, and the coefficient of planar anisotropy (Δr) were determined. As part of the preliminary tests, the surface roughness of sheet metal was also measured on the T1000 Hommel Tester profilometer (JENOPTIK Optical Systems GmbH, Berlin, Germany). The arithmetic mean surface roughness Ra was determined on a measuring section of 4.2 mm for three orientations relative to the RD: 0°, 45°, and 90°.

### 2.2. Formability Testing

The formability of sheets was determined using the Erichsen method (which allows for determining the material’s resistance to cracking in biaxial tension) in accordance with the recommendations of the EN ISO 20482 standard [41]. Moreover, the Fukui method (determining the technological properties of the sheet) in accordance with the requirements of the JIS Z 2249 standard [42] was also analyzed. For the Erichsen test, samples with dimensions of 90 mm × 90 mm were used.

The blank holder force and the spherical-ended punch speed were 10 kN and 3 mm/min, respectively. During the test, graphite lubricant was used on the surface of the sheet in contact with the punch to minimize the effect of friction. The test was carried out until a crack appeared through the sheet. The Erichsen number IE was determined based on the distance traveled by the punch to the crack. The Fukui test was carried out at a constant punch speed of 50 mm/min without the use of a blank holder force or lubricant. This test was carried out on a conical die until a crack appeared on the surface of the sample.

During the test, both the diameter of the discs before testing and the largest and smallest diameters of the conical drawpieces were taken into account for the analysis. On their basis, the average diameter of the conical drawpiece was determined. The Fukui coefficient η_F_ was calculated as the ratio of the average diameter of the conical drawpiece to the initial diameter of the disc. The tests were carried out on an Erichsen universal machine for testing sheet metals (model 142-40) (Elma, Gliwice, Poland).

### 2.3. Single-Point Incremental Forming Methodology

**Figure 1:**
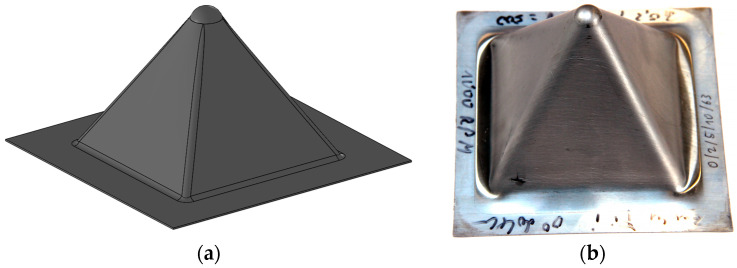
(**a**) Illustration of designed pyramid shape and (**b**) photography of sample drawpiece.

The Zn-Cu-Ti zinc alloy sheets were subjected to SPIF tests on a three-axis CNC BF30 milling machine (Stürmer Maszyny Sp. z o.o., Kostrzyn, Poland) equipped with a FANUC oi-MD controller. The machine was also equipped with an ISF table with a working area of 100 × 100 mm and a height of 150 mm. The tests were performed on 130 × 130 mm flat sheets. The length of the base side of the pyramid-shaped drawpieces (Figure 1) depended on the diameter of the working tool. This length was 86 mm for a 12 mm diameter tool and 78 mm for a 20 mm diameter tool. The conditions of the SPIF experiments are presented in Table 2. The sample orientation was achieved by rotating the input material (flat sheet) with an angle of 45 degrees. In all cases, a spiral forming trajectory with a constant vertical step size and constant forming angle was used. Graphite grease was used as a lubricant in the study.

### 2.4. Analysis of Drawpieces After ISF

Following incremental sheet forming, the drawpieces were subjected to a comprehensive analysis encompassing shape deviation, material properties, and surface roughness. The actual values of the wall angle were also determined. For this purpose, the drawpieces were subjected to three-dimensional scanning with a Zeiss ATOS CORE 200 3D scanner (Lenso Sp. z o.o., Poznań, Poland). On the basis of these measurements, the values of the wall angle to the base of the drawpiece, the profile of the wall in its axis for two adjacent walls (with an axis parallel and perpendicular to RD), and the size deviation in the circumferential direction of the inner radius of the side edge of the drawpiece in relation to the radius of the punch were determined.

The influence of the ISF process parameters on mechanical properties and surface roughness was analyzed for drawpieces with a wall angle of 60°. The test samples were precisely sectioned along the wall axis for two distinct wall orientations relative to the rolling direction (RD): parallel (0°) and perpendicular (90°). The gauge width and gauge length were 1.6 mm and 15 mm, respectively. The tensile test was conducted at a constant strain rate of 5 × 10^−3^ s^−1^. Surface roughness was measured for both the outer and inner walls of the drawpieces, in the directions longitudinal and transverse to the wall axis.

### 2.5. Analysis of Variance

In order to determine the significant factors influencing the surface roughness of the inner and outer surfaces of the pyramid-shaped drawpieces, analysis of variance was performed. A model was created that took into account the influence of quantitative inputs (step size, feed rate, and tool rotational speed), categorical input (side of the drawpiece) on the values of the arithmetic mean surface roughness Ra measured on the inner and outer sides of the drawpieces. The significance of the input parameters was assessed using the Fisher test at the level of significance α = 0.05. The aim of the ANOVA modeling was to obtain the optimal response of the polynomial regression model using the response surface methodology.

## 3. Results and Discussion

### 3.1. Mechanical Properties of Test Material

**Figure 2:**
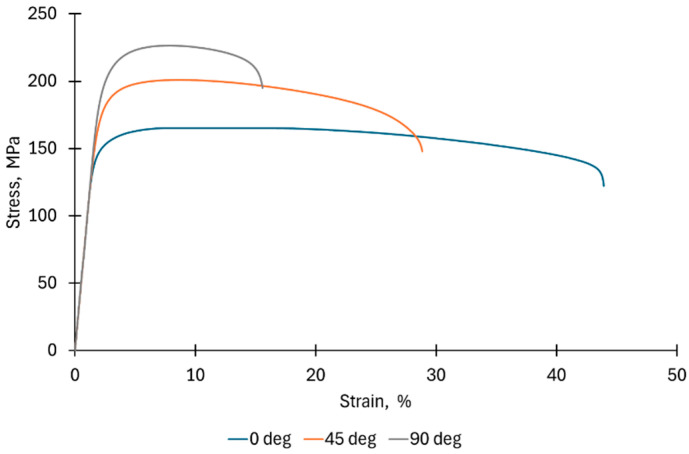
Stress-strain curves of Zn-Cu-Ti sheet for different sample orientations with respect to RD.

Figure 2 shows stress-strain curves for the test material. They show significant differences in the properties of the Zn-Cu-Ti alloy sheet depending on the direction of cutting the samples from the sheet metal (Table 3). The highest ultimate tensile strength (UTS) and the lowest elongation (A) were found for the 90° sample orientation. On the other hand, the sample cut parallel to the RD was characterized by the highest elongation A and the lowest yield strengths (YSs), with the lowest strength properties. This is related to the HCP structure of the zinc alloy, which contributes to the strong plastic anisotropy of the material. The anisotropy coefficient (r-value) for the test material was different depending on the sample orientation. The highest r-value was found for the 90° direction (0.194), and the lowest for the 0° direction (0.163). A low average value of the normal (Lankford) anisotropy coefficient, not exceeding 0.2, is not conducive to the implementation of deep drawing or redrawing processes. There is a high risk of too much necking during the forming of the material, and consequently its cracking [43]. However, a low r-value may be beneficial during bulging of the sheet metal [44]. Additionally, the high tendency of the material to deformation in the thickness direction may have a beneficial effect on the incremental forming process, in which local deformation of the material occurs through stretching and bending [45]. The value of the degree of planar anisotropy Δr equal to −1.5 suggests that during the forming of axisymmetric drawpieces using the conventional SMF method, geometric irregularity of the edge of the drawpiece (earing defect) may appear, which is particularly important during deep drawing. For incremental forming, where the sheet flange is fixed, the effect of anisotropy on the appearance of the side edge of the drawpiece may be negligible. However, strong anisotropy may be significant in the case of material flow in the deformation zone.

Table 4 presents the results of the measurement of the drawability coefficients using two methods, Erichsen and Fukui. The Erichsen test allows for the assessment of the sheet metal resistance to cracking in the case of biaxial stretching on the punch face. The value of the Erichsen number IE (9.6) corresponds to the results obtained for steel sheets with good drawability. The Fukui method, on the other hand, is a test determining the technological properties of sheets. In contrast to the Erichsen test, the influence of the sheet metal thickness on the Fukui coefficient η_F_ is minimal. The lower the η_F_ value, the better the sheet metal’s ability to be formed. For the test material, its value was equal to 0.818.

### 3.2. SPIF of Zn-Cu-Ti Zinc Alloy Sheet

#### 3.2.1. The Limit-Forming Angle

**Figure 3:**
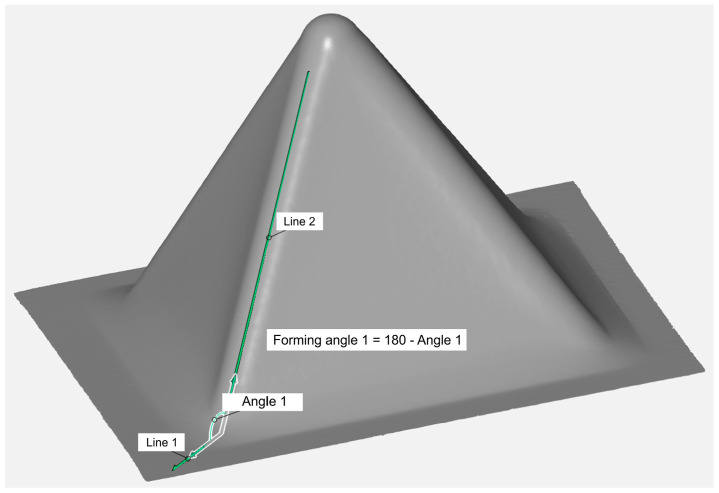
Method of measuring the limit-forming angle of the pyramid-shaped drawpiece.

**Figure 4:**
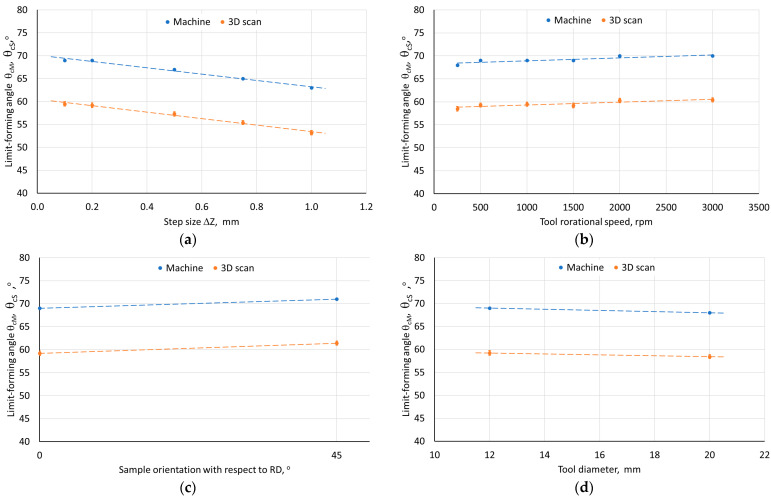
The influence of the (**a**) step size, (**b**) tool rotational speed, (**c**) sample orientation with respect to RD, and (**d**) on the limit-forming angle of the pyramid-shaped drawpieces.

Figure 3 shows the method of measuring the actual angle of inclination of the pyramid-shaped drawpiece wall. In the tests, constant values of the forming angle were used along the entire height of the drawpiece. The measurement of angle was taken between the line lying on the edge between the walls and the drawpiece base. The limit-forming angle Θ_c_ was defined as the maximum forming angle of a pyramid-shaped drawpiece at which fracture did not occur; any angle exceeding this value resulted in destruction of the drawpiece. The values of the limit-forming angle Θ_c_ for the analyzed ISF conditions are presented in Table 5. It was found that with the increase of the step size, the limit-forming angle value decreased (Figure 4a). This may be related to the increase of stresses in the material, resulting from the increase of the unit step size value [45], which caused the formation of distinct grooves on a certain area of the internal surface with an axis perpendicular to the RD. This phenomenon is discussed in more detail in Section 3.2.3 and Section 3.2.4 in the context of the orientation of the sample in the SPIF process.

Another factor important in terms of sheet formability in the ISF process is the tool rotational speed, especially at lower values of the step size and feed rate. It was observed that the increase in tool rotational speed from 250 rpm to 2000 rpm during SPIF of Zn-Cu-Ti sheets contributed to the increase in the actual value of the limit-forming angle from 58.5° to 60.2° (Figure 4b). The heat is generated by friction between the workpiece and the tool due to the high-speed rotary movement of a tool and a local increase in temperature contributing to the improvement of the material plasticity [46,47]. For feed rate, however, no significant difference in the value of the limit-forming angle was found despite the increase in the mechanical properties of the drawpiece.

**Figure 5:**
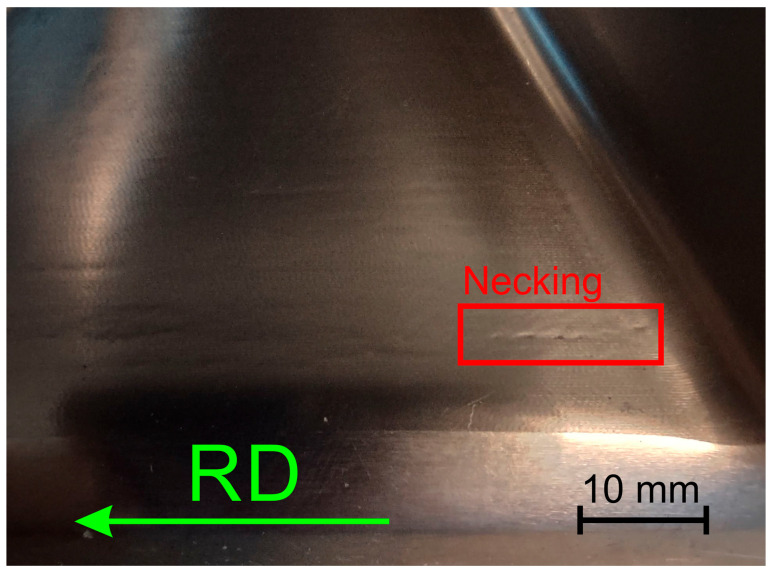
A necking visible on the inner surface of the drawpiece wall parallel to RD.

The ISF process parameters influencing the θ_c_-value were also the orientation of the sample relative to the rolling direction (Figure 4c) and the tool diameter (Figure 4d). In the first case, the change in sample orientation from 0° to 45° contributed to the increase in the value of the limit-forming angle. This was related to the large difference in mechanical properties in the sheet plane, especially between the orientations of 0° and 90°. On the drawpiece wall parallel to the sheet RD, cracks appeared much earlier than in the case of the wall oriented perpendicular to the RD. For the sample cut parallel to the RD, material discontinuities in the form of grooves appeared along a certain length of the wall on the inner surface of the drawpiece wall parallel to the RD (Figure 5). This was not observed on the wall perpendicular to the RD. Grooves were located at the rounding of the side edge of the drawpiece and did not occur or occurred with lower intensity in the further part of the wall. The presence of grooves is often associated with the so-called incipient necking and is closely related to the ratio of the initial sheet thickness to the radius of the forming tool, t_0_/R [48], which in the analyzed case was equal to 0.11. The observed grooves were the site of initiation of material cracking, which propagated across the wall of the drawpiece.

For the sample cut at an angle of 45° to the RD, the presence of areas of unstable material flow was also found on all walls of the drawpiece along its entire circumference. The largest number of grooves on the surface was observed, similarly to the previous case, at the rounding of the side edge of the drawpiece, immediately after changing the direction of the tool movement. The grooves for orientation 45° were formed at a higher degree of deformation (wall angle) than in the case of the samples with orientation 0°.

The tool diameter had the opposite effect on the formability of the Zn-Cu-Ti sheet than the orientation of the samples. The use of a 20 mm diameter tool contributed to a decrease in the limit-forming angle (Figure 4d), which can be associated with an increase in the tool-workpiece contact area and an increase in the amount of contact forces [49]. Moreover, the use of a tool with a diameter of 12 mm promotes greater local heating [46] and high strain accumulation, which leads to better formability [49,50]. Similarly to the case of the 12 mm diameter tool, the occurrence of localized necking was also observed for the 20 mm tool.

#### 3.2.2. Profile of the Wall of the Drawpieces

**Figure 6:**
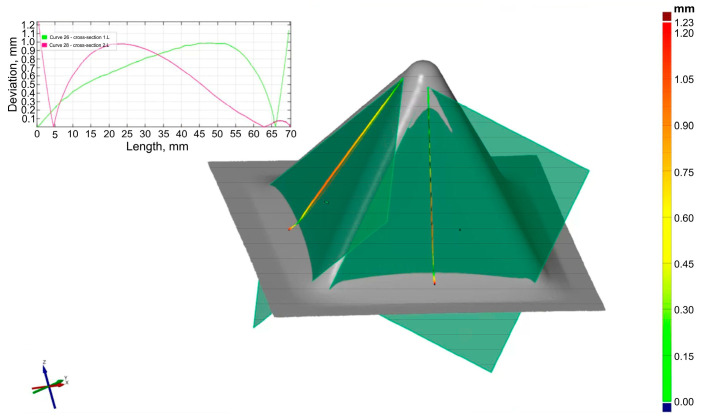
Method of determining the profile of the pyramid wall in its axis.

**Figure 7:**
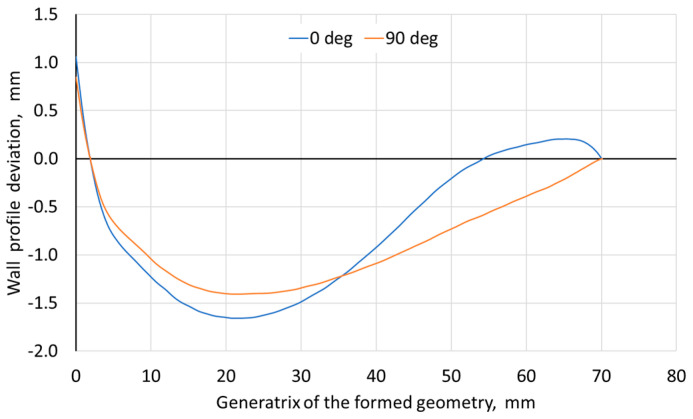
Profile of the pyramid wall along its axis for two orientations relative to RD; forming conditions: D = 12 mm, ΔZ = 0.2 mm, f = 500 mm/min, n = 3000 rpm.

Figure 6 shows how to determine the profile of the drawpiece wall in its axis. This allowed us to present the deflection of the wall after forming in relation to the theoretical flat surface (Figure 7). Analyses were performed for a wall angle equal to 60°. The orientation of the drawpiece wall in relation to the RD was taken into account.

**Figure 8:**
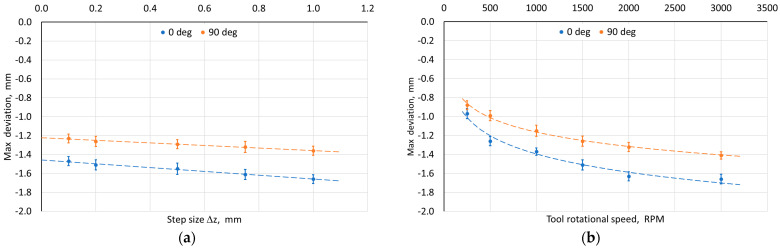

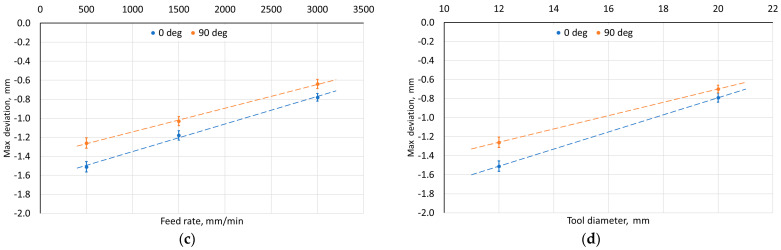
Influence of (**a**) step size, (**b**) tool rotational speed, (**c**) feed rate, and (**d**) tool diameter on the maximum deviation of the measured wall profile from the ideal profile depending on measurement orientation relative to RD.

In all analyzed cases, a greater deviation from the wall plane (tangent to the side edges of the drawpiece) was found for the pyramid wall, the axis of which coincided with the RD. This indicates a certain influence of the anisotropy of the workpiece properties on the final shape of the drawpiece produced using SPIF. It was found that with the increase of the step size (Figure 8a) and rotational speed (Figure 8b), the maximum deviation of the measured wall profile from the ideal profile also increased. However, the step size had a significantly smaller effect than the tool rotational speed. The increase in the wall profile deviation with the increase of the step size may be related to a smaller local deformation, and therefore a larger stress gradient in the material and higher residual stresses [51].

Despite the increase in the tool rotational speed and, consequently, in the frictional heat caused by sliding the tool against the workpiece, the maximum wall deviation also increased (Figure 8b). On the other hand, the increase in the feed rate (Figure 8c) and tool diameter (Figure 8d) had a positive effect on reducing the deviation. For the convex areas, the positive deviation (in the outer direction of the drawpiece) was in the range of 0.03 to 0.13 mm and was similar in character to the negative deviation (in the inner direction of the drawpiece). For the test material, it can be stated that all tested factors influence the wall profile deviation significantly. However, the increase in feed rate and tool diameter with a simultaneous decrease in the step size and its feed rate should contribute to reducing the wall profile deviation in its axis.

#### 3.2.3. Radius of the Side Edge of the Drawpiece

**Figure 9:**
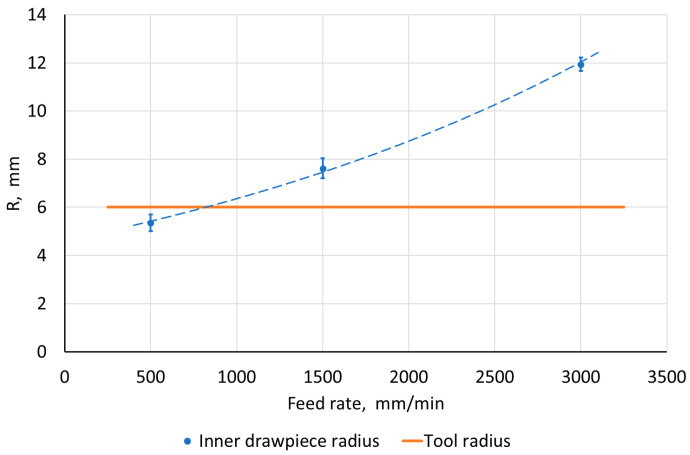
The influence of the feed rate on the representation of the radius R of the inner edge of the drawpiece.

The work analyzed the influence of SPIF parameters on the value of the inner radius of the side edge of a pyramid-shaped drawpiece. For the step size, tool rotational speed, tool diameter, and sample orientation, no significant differences were found between the tool radius and the radius of the drawpiece edge. They did not exceed 10% and were practically independent of the mentioned SPIF parameters. However, in the case of feed rate, a significant influence of this parameter on the value of the edge radius was found. For this purpose, an optical scan of the drawpiece (Figure 6) was used, based on which the fillet radii were measured. It was found that with the increase of the feed rate, the accuracy of the obtained radius inside the drawpiece decreased (Figure 9). Its lowest value was obtained in the case of a feed rate of 500 mm/min and it was smaller than the tool radius, which can be related to the springback phenomenon. On the other hand, the reason for the significant increase in the value of the fillet radius of the drawpiece edge can be attributed to the method of controlling the tool movement path. In the analyzed cases, the feed rate was constant over the entire length of the path, which contributed to a certain inertia when changing the direction of its movement at higher feed rates. Probably, the use of a reduced optimal feed rate in the edge area should contribute to the improvement of the accuracy of mapping the side edge of the pyramid-shaped drawpiece.

#### 3.2.4. SPIF-Induced Mechanical Properties of the Drawpieces

**Figure 10:**
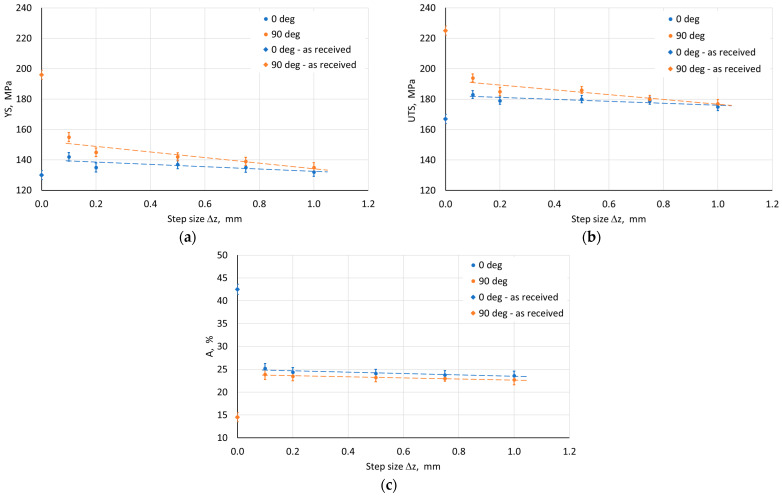
Effect of step size on the parameters (**a**) YS, (**b**) UTS, and (**c**) elongation A measured on walls with axes parallel (0°) and perpendicular (90°) to RD.

**Figure 11:**
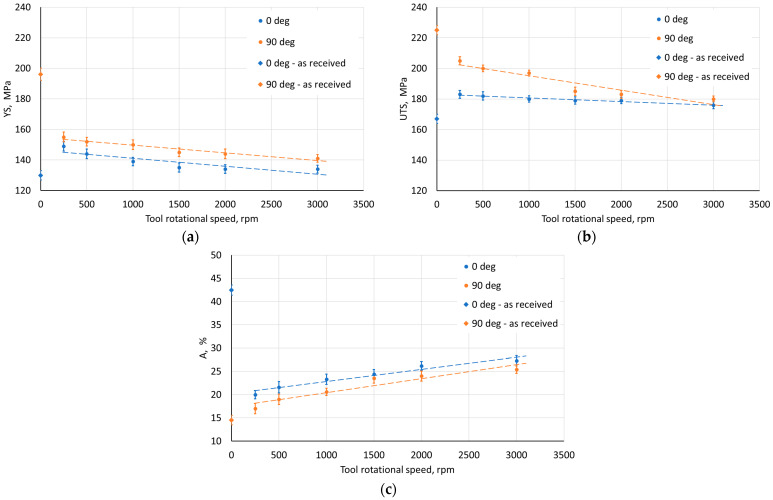
Effect of tool rotational speed on the parameters (**a**) YS, (**b**) UTS, and (**c**) elongation A measured on walls with axes parallel (0°) and perpendicular (90°) to RD.

**Figure 12:**
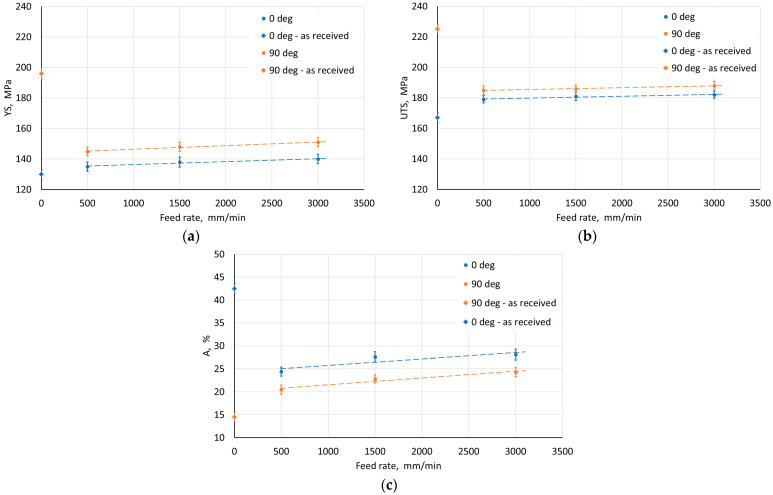
Effect of feed rate on the parameters (**a**) YS, (**b**) UTS, and (**c**) elongation A measured on walls with axes parallel (0°) and perpendicular (90°) to RD.

**Figure 13:**
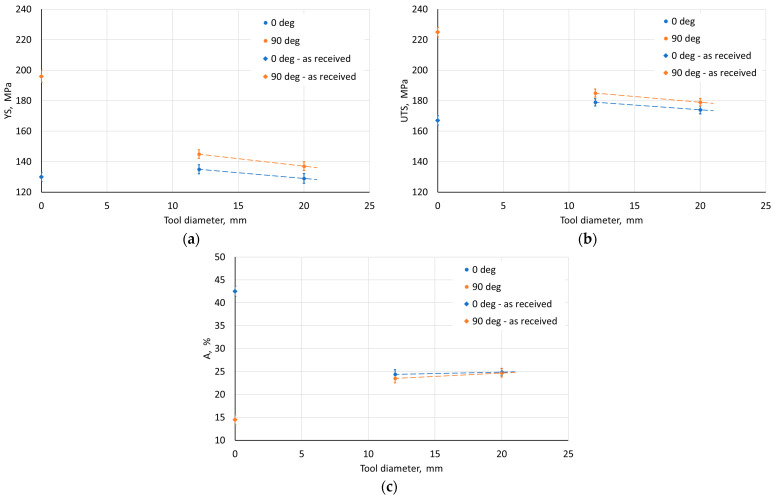
Effect of tool diameter on the parameters (**a**) YS, (**b**) UTS, and (**c**) elongation A measured on walls with axes parallel (0°) and perpendicular (90°) to RD.

**Figure 14:**
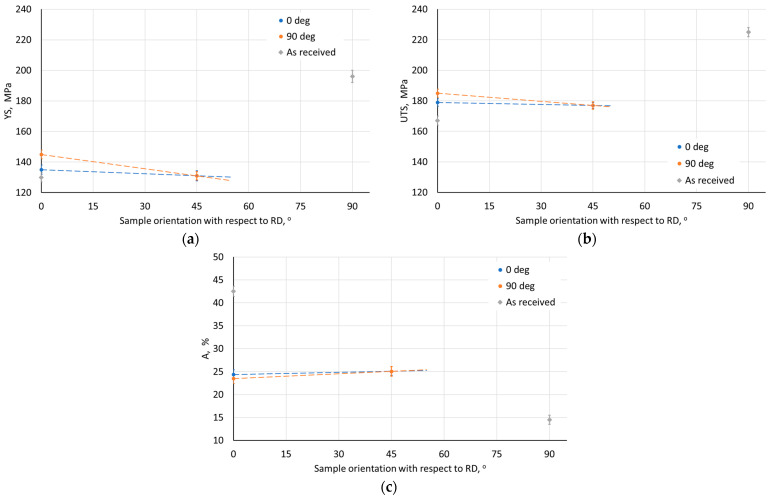
Effect of sample orientation with respect to RD on the parameters (**a**) YS, (**b**) UTS, and (**c**) elongation A measured on walls with axes parallel (0°) and perpendicular (90°) to RD.

The influence of ISF process parameters on mechanical properties was analyzed for drawpieces with a wall angle of 60°. The test samples were cut along the wall axis in the direction parallel (0°) and perpendicular to the RD (90°). The width of the measurement base was 1.6 mm, and its length was 15 mm. Regardless of the applied SPIF conditions, a difference in mechanical properties was found between adjacent walls (Figure 10, Figure 11, Figure 12, Figure 13 and Figure 14). Samples cut from walls with an axis perpendicular to the RD were characterized by higher strength properties (Figure 10a,b) and lower plasticity (Figure 10c) in comparison to samples cut from walls with an axis parallel to the RD. This means that the anisotropic character of the workpiece material has a significant effect on the properties of the drawpieces. Although the difference in mechanical parameter values for directions 0° and 90° decreased after SPIF.

The step size had an unfavorable effect on the sheet metal properties. As it increased, the YS and UTS of the material decreased (Figure 10a,b). This may be due to the greater distance between forming marks resulting from more intense unit deformation at larger step sizes. The tool rotational speed also has an unfavorable effect on the material strength of the drawpiece, reducing it with increasing speed (Figure 11a,b). This type of situation was also noticed by Lu et al. [52] and Ilyas et al. [53]. The reduction in strength with simultaneous increase in plasticity is associated with friction-induced heating of the workpiece material. Moreover, with increasing temperature, the reconstruction of the material structure occurs to some extent during SPIF, which is more intensive the higher the tool rotational speed. On the other hand, the negligible effect of the feed rate on the analyzed mechanical parameters (Figure 12) was related to a slight increase in frictional heat at higher tool speeds.

Figure 13 shows the effect of the tool diameter on selected mechanical properties of the drawpieces determined for walls with axes parallel (0°) and perpendicular (90°) to the RD of the Zn-Cu-Ti sheet. The use of a tool with a larger diameter adversely affects the strength of the material (Figure 13a,b), while its plasticity slightly increases (Figure 13c). The presented effect can be related to the decrease in hydrostatic compression, responsible for the deformability of the material in SPIF with the increase of the tool diameter [54].

The strength of the drawpiece made from the sample cut at an angle of 45° to the RD was lower than for the 0° orientation (Figure 14). This is related to the properties of the sheet metal, the values of which for the 45° orientation were between the values for the 0° and 90° orientations (Table 3). It should be noted that the 0° and 90° orientations for the sample with the 45° orientation corresponded to adjacent walls. Therefore, average values of mechanical parameters were determined for this sample orientation (45°). However, the difference between the values of the analyzed mechanical properties for this sample did not exceed 3%.

Despite the decrease in YS and UTS (Figure 14a,b), with a simultaneous increase in plasticity (Figure 14c), a decrease in the so-called ‘plasticity reserve’ (YS/UTS) [18] was also observed in the material for the orientation of 45° (0.740) in relation to the orientation of the wall axis relative to RD 0° (0.754) and 90° (0.784). This may partly explain the better formability of the sample with the 45° orientation. Moreover, the ‘plasticity reserve’ values were lower than for the workpiece in the as-received state.

#### 3.2.5. Surface Roughness

##### Experimental Analysis

**Figure 15:**
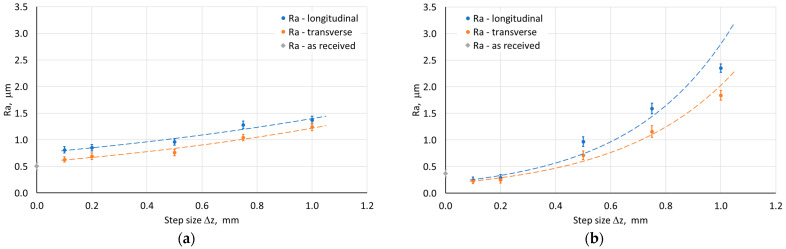
Influence of step size on the average roughness Ra of (**a**) outer and (**b**) inner surface of the drawpiece; SPIF parameters: D = 12 mm, f = 500 mm/min, n = 1500 rpm.

**Figure 16:**
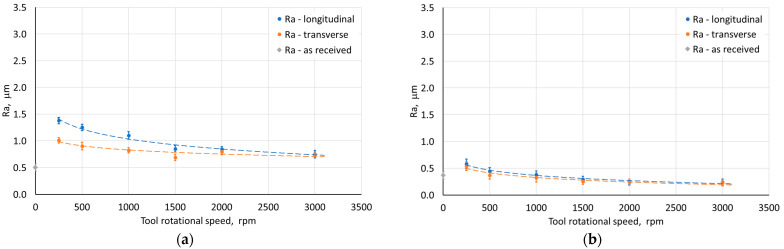
Influence of tool rotational speed on the average roughness Ra of (**a**) the outer and (**b**) inner surface of the drawpiece; SPIF parameters: D = 12 mm, f = 500 mm/min, Δz = 0.2 mm.

**Figure 17:**
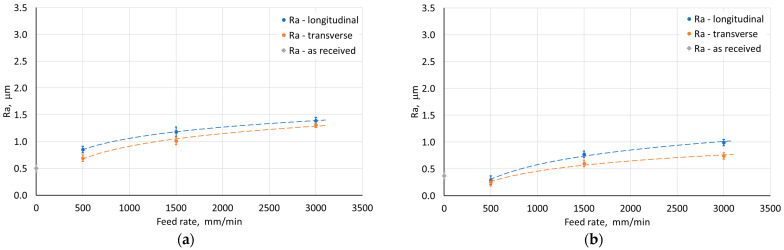
Influence of feed rate on the average roughness Ra of (**a**) the outer and (**b**) the inner surface of the drawpiece; SPIF parameters: D = 12 mm, n = 1500 rpm, Δz = 0.2 mm.

**Figure 18:**
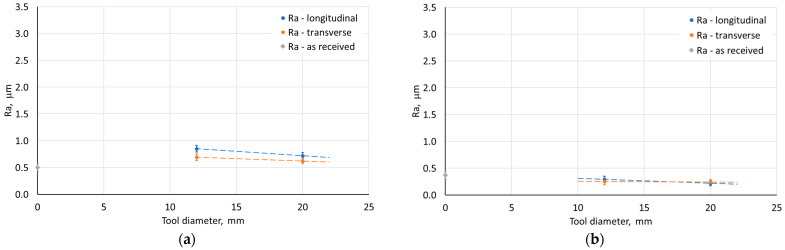
Influence of tool diameter on the average roughness Ra of (**a**) the outer and (**b**) the inner surface of the drawpiece; SPIF parameters: f = 500 mm/min, n = 1500 rpm, Δz = 0.2 mm.

**Figure 19:**
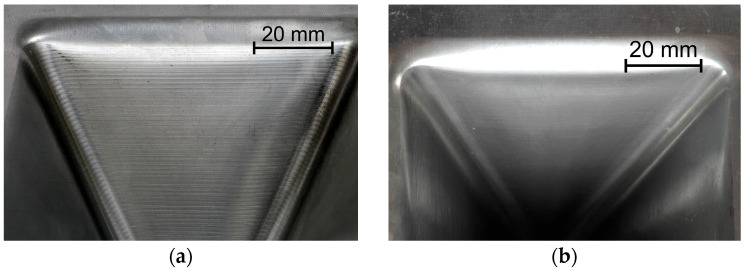
The appearance of the inner surface of the pyramid-shaped drawpiece for a step size of (**a**) 1 mm and (**b**) 0.2 mm; SPIF parameters: D = 12 mm, f = 500 mm/min, n = 1500 rpm.

Figure 15, Figure 16, Figure 17 and Figure 18 show the influence of the analyzed SPIF parameters on the arithmetic mean surface roughness Ra of the inner and outer walls of the drawpieces. The tests were performed for a drawpieces wall angle of 60°. For the step size, the average roughness increased, which was strongly related to the presence of cyclic grooves, especially on the inner surface (Figure 19a). Too large a value of step size may result in the formation of cyclic grooves in the drawpiece surface as a result of the interaction of the tool tip with the surface of the deformed sheet metal. The presence of forming marks (grooves) affected the arithmetic mean surface roughness Ra value measured in the longitudinal and transverse directions relative to the wall symmetry axis. However, reducing the step size caused the successive forming marks to overlap, gradually reducing the peaks and valleys on the sheet metal surface [55], causing the surface roughness to decrease significantly to a step size of 0.2 mm (Figure 15 and Figure 19b). For a step size of 0.1 mm, the measured Ra values were similar. Hence, only the 0.2 mm step was taken into account in further analyses.

**Figure 20:**
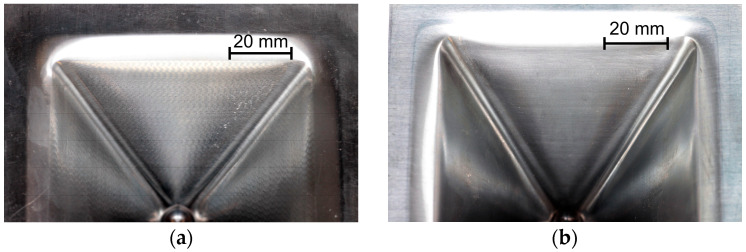
The appearance of the inner surface of the pyramid-shaped drawpiece for a tool rotational speed of (**a**) 250 rpm and (**b**) 1000 rpm; SPIF parameters: D = 12 mm, f = 500 mm/min, Δz = 0.2 mm.

The opposite effect than for the step size was obtained, however, in the case of the variable tool rotational speed at a constant feed rate (Figure 16). With the increase of the tool rotational speed, the Ra value decreased (Figure 16). For low tool rotational speeds (250 rpm), characteristic “fish scale” marks were observed on the inner surface of walls (Figure 20a). With the increase of the rotational speed, the tool marks gradually disappeared (for n/f = 2), as shown in Figure 20b. At the tool rotational speed of 1500 rpm (n/f = 3), the value of the arithmetic mean surface roughness Ra stabilized (Figure 16b). Interestingly, despite the presence of visible forming marks on the inner surface of the wall, the outer surface was characterized by higher Ra values. This could be related to the presence of micro-cracks on outer surface originating from tensile stresses generated during the tool pressure on the sheet material [45]. A similar effect was also obtained in the work of Trzepieciński et al. [56], where higher values of the roughness parameters and ‘orange peel’ patterns were found for the outer surface of the conical drawpiece.

For variable feed rate at constant tool rotational speed, an inverse character of the change in the Ra parameter value was found with the change in feed rate (Figure 17). This is related to the n/f ratio, where at its low value (high linear velocity) there is an increase in the arithmetic mean surface roughness resulting from the presence of distinct forming traces from the tool impact. On the other hand, increasing the tool diameter contributed to a decrease in the Ra parameter (Figure 18). Using a tool with a larger diameter at the same step size causes the forming marks to overlap more, leading to smoothing the surface of the drawpiece wall.

Compared to the sheet roughness, the Ra values for the outer wall of the drawpiece were higher, regardless of the parameters used. However, for the inner wall roughness of the drawpiece, the influence of the parameters was different. When the step size was less than 0.5 mm, the tool speed exceeded 1000 rpm, the feed was 500 mm/min, and both punch diameters were used, the inner surface of the drawpiece showed lower Ra values than the sheet. In all other cases, with the ISF parameters used, the roughness of inner surface of the pyramidal drawpiece was higher.

##### Analysis of Variance

Table 6 presents the basic statistics of the ANOVA model concerning the arithmetic mean surface roughness Ra measured in the transverse direction. The F-value of 33.25087 means that the model is significant. The parameters that are most strongly correlated with the arithmetic mean surface roughness Ra are step size and side of the drawpiece (inner and outer) on which the roughness parameter was measured. Based on backward elimination regression, the tool rotational speed (C) and tool diameter (D) were excluded from the final model. Their *p*-values were greater than 0.05.

The capability of the model determined by the coefficient of determination at a 95% confidence interval is R^2^ = 0.7808, which shows satisfactory agreement with experimental data. The signal-to-noise ratio (adequacy precision) was equal to 20.67, which is significantly greater than 4, thus indicating adequate signal.

The equation, which describes the arithmetic mean surface roughness Ra measured in the transverse direction, is given in Equation (1) with the coded factors:

(1) Ra (transverse)=1.19186+0.51797×A+0.263487×B+−0.169375×E

**Figure 21:**
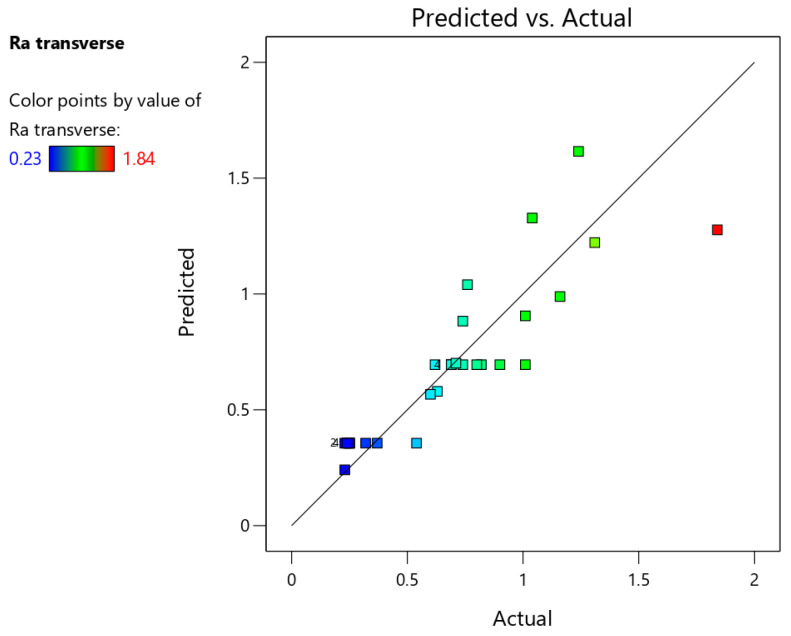
Predicted vs. actual response for the arithmetic mean surface roughness Ra measured in the transverse direction.

Figure 21 shows a comparison of actual and predicted values of surface roughness parameter Ra. The proportional distribution of points along the regression line confirms the strong statistical correlation between predicted and actual values of the arithmetic mean surface roughness Ra measured in the transverse direction.

**Figure 22:**
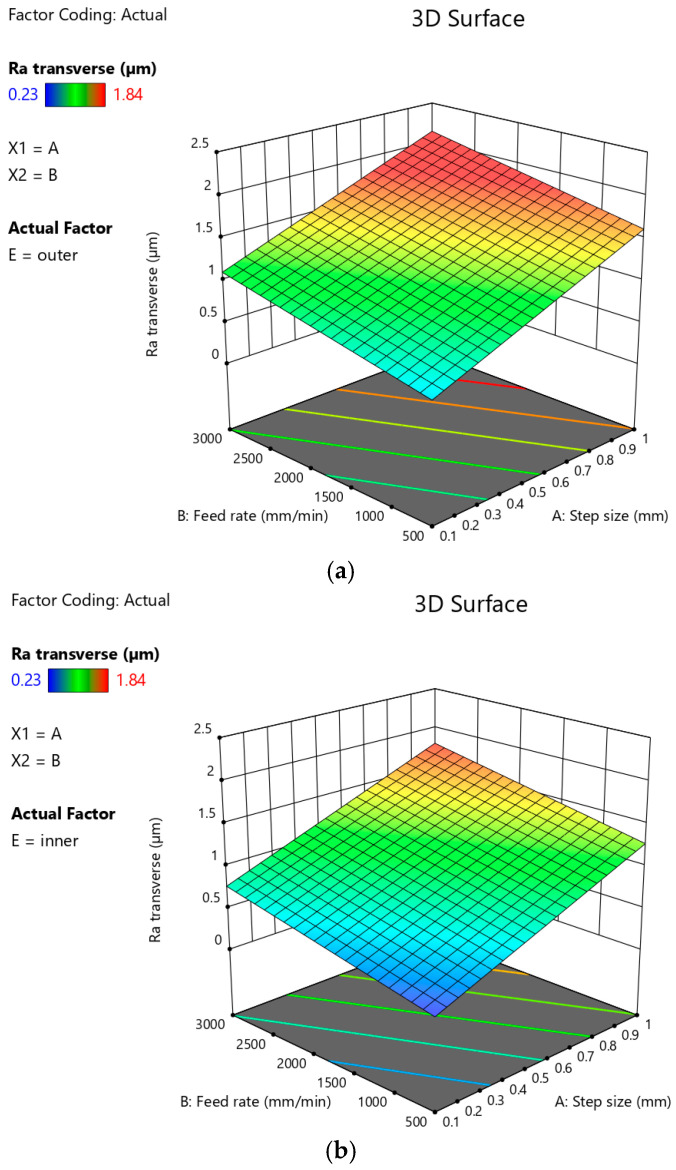
Plots showing the effect of step size and feed rate on the arithmetic mean surface roughness Ra measured on (**a**) the outer and (**b**) the inner side of the drawpiece in the transverse direction.

For both the outer (Figure 22a) and inner (Figure 22b) sides of the drawpiece, an increase in the arithmetic mean surface roughness Ra value was observed with the increase in step size. On the other hand, a simultaneous decrease in the feed rate causes a decrease in the Ra value measured in the transverse direction. In this way, the highest roughness of both surfaces of the drawpiece (outer and inner) is caused by high values of the feed rate and simultaneously high values of the step size.

Table 7 presents the basic statistics of the ANOVA model concerning the arithmetic mean surface roughness Ra measured in the longitudinal direction. The F-value of 21.08243 means that the model is significant. The parameters that are most strongly correlated with the arithmetic mean surface roughness Ra are step size and side of the drawpiece (inner and outer) on which the roughness parameter was measured. Based on backward elimination regression, the tool diameter (D) was excluded from the final model.

The capability of the model determined by the coefficient of determination at a 95% confidence interval is R^2^ = 0.7575, which shows satisfactory agreement with experimental data. The signal-to-noise ratio (adequacy precision) was equal to 18.04, which is significantly greater than 4, thus indicating adequate signal.

The equation, which describes the arithmetic mean surface roughness Ra measured in the longitudinal direction, is given in Equation (2) with the coded factors:

(2) Ra (longitudinal)=1.42506+0.665926×A+0.302801×B−0.247153×C−0.195313×E

**Figure 23:**
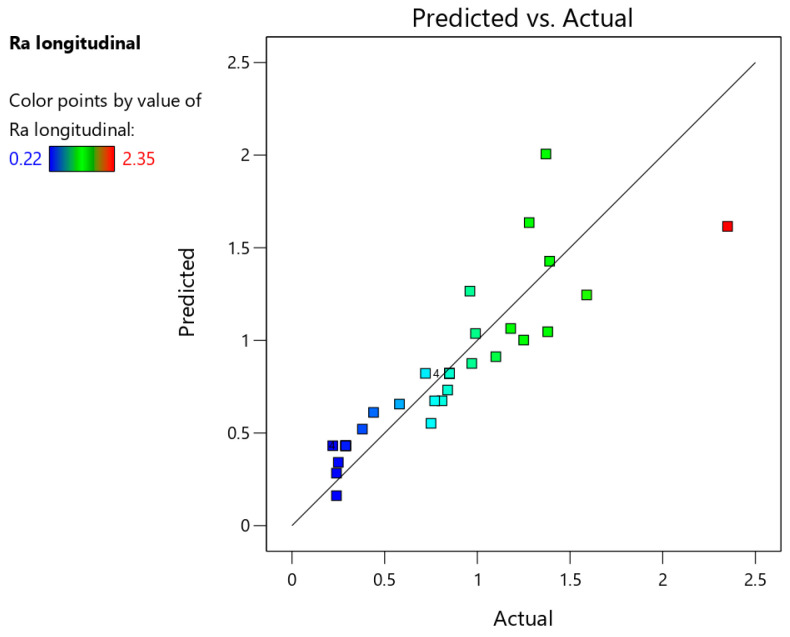
Predicted vs. actual response for the arithmetic mean surface roughness Ra measured in the longitudinal direction.

Figure 23 shows a comparison of actual and predicted values of surface roughness parameter Ra. The proportional distribution of points along the regression line confirms the strong statistical correlation between predicted and actual values of the arithmetic mean surface roughness Ra measured in the longitudinal direction.

**Figure 24:**
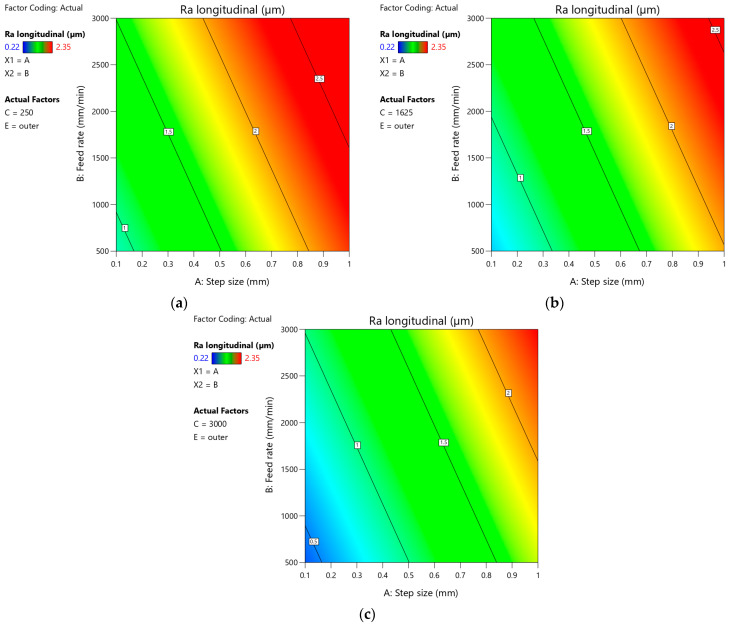
Plots showing the effect of step size and feed rate on the arithmetic mean surface roughness Ra measured on the outer side of the drawpiece in the longitudinal direction for (**a**) minimum value of tool rotational speed (250 rpm), (**b**) average value of tool rotational speed (1625 rpm), and (**c**) maximum value of tool rotational speed (3000 rpm).

**Figure 25:**
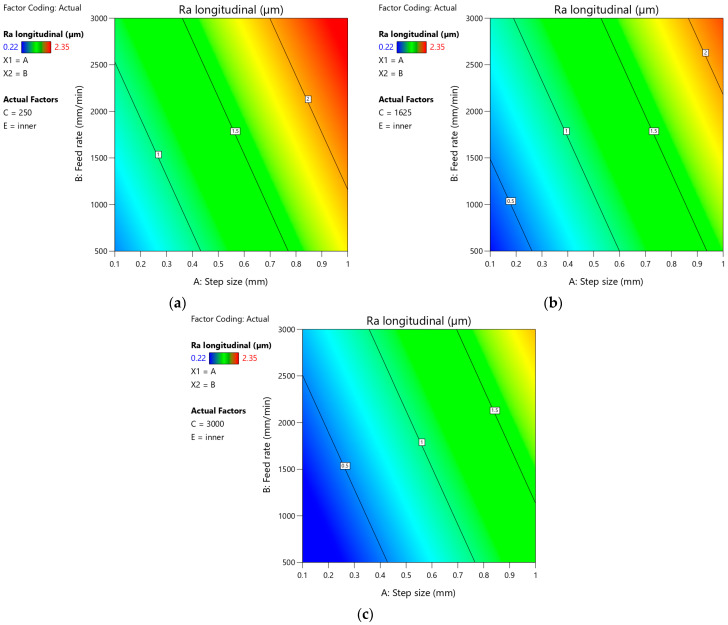
Plots showing the effect of step size and feed rate on the arithmetic mean surface roughness Ra measured on the inner side of the drawpiece in the longitudinal direction for (**a**) minimum value of tool rotational speed (250 rpm), (**b**) average value of tool rotational speed (1625 rpm), and (**c**) maximum value of tool rotational speed (3000 rpm).

Figure 24a–c presents plots showing the effect of step size and feed rate on the arithmetic mean surface roughness Ra measured on the outer side of the drawpiece in the longitudinal direction for the minimum, average, and maximum values of tool rotational speed, respectively. It was found that the character of the influence of tool rotational speed on the arithmetic mean surface roughness Ra is similar on all plots. The angles of inclination of individual contours are similar. With the increase of feed rate and step size, the value of the Ra parameter increases. This is consistent with the observations noted in Figure 22b. The above conclusion can also be applied to Figure 25. However, the SPIF with the highest value of tool rotational speed (Figure 25c) provides the lowest arithmetic mean surface roughness Ra in the analyzed range of variation of step size and feed rate values. In turn, the forming with the lowest value of tool rotational speed (Figure 24a and Figure 25a) produces the highest roughness on both sides of the drawpiece.

## 4. Conclusions

The influence of variable SPIF process parameters on the value of basic mechanical parameters, maximum deviation of the measured wall profile from the ideal profile, limit-forming angle, and surface roughness of HCP Zn-Cu-Ti pyramid-shaped drawpieces was analyzed in this work. The following conclusions can be drawn based on the results obtained:

- Zn-Cu-Ti alloy sheet is characterized by high anisotropy of mechanical properties and plastic anisotropy (determined by the Lankford coefficient). This feature significantly affects the behavior of the material during SPIF, contributing to a lower wall angle for the 0° orientation of the sample in relation to the 45° orientation.
- The large difference in the properties of the workpiece material between the orientations 0° and 90° relative to the RD causes the creation of transverse grooves during SPIF only on the drawpiece walls perpendicular to the sheet RD, which are the sources of material cracking. For the orientation 45°, transverse grooves also appear, but at higher values of the drawpiece wall angle.
- The SPIF process parameters had a different effect on the mechanical properties of the drawpieces. Regardless of the SPIF process conditions, however, there was a significant difference in the basic mechanical properties measured in the walls oriented parallel and perpendicular to the RD. The drawpieces were characterized by higher values of strength parameters being found for the walls with an orientation of 90° in relation to the RD. Higher elongation after rupture was observed for the walls with an axis parallel to the RD.
- All the factors analyzed had an impact on the value of the limit-forming angle of the drawpiece wall. The greatest impact was found in the case of the step size, where the increase in its value was accompanied by a decrease in the value of the limit-forming angle. For the remaining parameters, their influence on the limit-forming angle was moderate, although not insignificant.
- Both the step size and the tool rotational speed contribute to the increase of the maximum wall profile deviation. However, the use of higher feed rates and a larger tool diameter caused its reduction. At the same time, the increase in feed rate caused a smaller reproduction of the rounding of the side edge of the drawpiece. At a feed rate of 3000 mm/min, the value of rounding was about two times larger than the tool radius.
- The surface roughness of the surface of the drawpiece walls varied depending on the SPIF process parameters. Higher values of arithmetic mean surface roughness Ra were found for the outer surface of drawpieces. One of the important parameters influencing the Ra value was the step size. High values of this parameter contributed to the formation of clear forming marks. On the other hand, the use of a smaller step size with a larger tool diameter caused the individual forming marks to overlap and reduced the surface roughness of the drawpiece wall.
- The use of low tool rotational speeds at constant feed rate contributed to the formation of distinct topography in the form of ‘fish scale” marks on the inner wall surface. Similar effects were observed at high feed rates, with a constant value of the tool rotational speed. It was found that at a ratio of tool rotational speed to feed rate (n/f ≥ 2), visible forming marks no longer appeared on the inner surface of the drawpiece.
- Based on the analysis of variance, it was found that the value of the arithmetic mean surface roughness Ra measured in the transverse direction significantly depends only on the step size, feed rate, and measurement side (inner, outer) on the drawpiece. For the longitudinal direction, the tool rotational speed also significantly determines the Ra value. For both measurement directions, it was found that with the increase of the step size and feed rate, the value of the Ra parameter increases.

In the context of ensuring large formability of Zn-Cu-Ti sheet in the SPIF process, the obtained results suggest using samples cut at an angle of 45° to the RD. This allows us to obtain the highest degree of deformation with simultaneous good mechanical properties of the SPIFed component. At the same time, the appropriate selection of the step size, feed rate, and tool rotational speed, when using tools with small radii, can additionally contribute to increasing the value of the limit-forming angle and the height of the drawpiece. A high ratio of tool rotational speed to feed rate and a small step size also ensure better surface quality on the inner and outer sides of the drawpiece. Optimal selection of SPIF process parameters can contribute to obtaining components with a high degree of deformation and high surface quality.

## Figures and Tables

**Table 1:** Chemical composition of ZnCuTi alloy (wt.%).

Zn	Cu	Ti
98.9	0.95	0.15

**Table 2:** Test conditions for SPIF of Zn-Cu-Ti sheet metal.

Sample Orientation (Relative to RD), °	Feed Rate f, mm/min	Tool Rotational Speed n, rpm	Tool Diameter D, mm	Step Size in Z-Direction Δz, mm
0, 45	500, 1000, 1500, 2000, 3000	250, 500, 1000, 1500, 3000	12, 20	0.1, 0.2, 0.5, 0.75, 1.0, 1.2

**Table 3:** Basic mechanical properties of Zn-Cu-Ti sheet.

Sample Orientation, °	YS, MPa		UTS, MPa		A, %		r-Value		Δr
	Mean Value	95% CI	Mean Value	95% CI	Mean Value	95% CI	Mean Value	95% CI	
0	130 ± 1.5	127–133	167 ± 1.1	164– 170	42.5 ± 1.56	41.4–43.6	0.163 ± 0.0021	0.158–0.168	−1.5
45	181 ± 2.1	178–184	204 ± 1.2	201–207	26.1 ± 0.95	24.8–27.6	0.180 ± 0.0012	0.177–0.183	
90	196 ± 3.6	192–200	225 ± 1.1	222–228	14.5 ± 0.67	13.5–15.5	0.194 ± 0.0015	0.190–0.198	
Average value	172	-	200	-	27.3	-	0.179	-	

**Table 4:** Results of the formability tests of Zn-Cu-Ti sheets.

Erichsen Number IE, mm	Fukui Coefficient η_F_
9.6 ± 0.15	0.818 ± 0.0061

**Table 5:** Limit-forming angle values depend on the ISF process conditions.

Tool Diameter D, mm	Step Size Δz, mm	Feed Rate f, mm/min	Tool Rotational Speed n, rpm	Sample Orientation According to the RD, °	Limit-Forming Angle θ_cM_, °	Limit-Forming Angle (Based on the 3D Scanning) θ_cS_, °
12	0.1	500	1500	0	69	59.5 ± 0.29
12	0.2	500	1500	0	69	59.2 ± 0.32
12	0.5	500	1500	0	67	57.3 ± 0.29
12	0.75	500	1500	0	65	55.4 ± 0.26
12	1.0	500	1500	0	64	54.2 ± 0.31
12	0.2	500	250	0	68	58.5 ± 0.32
12	0.2	500	500	0	69	59.3 ± 0.25
12	0.2	500	1000	0	69	59.5 ± 0.27
12	0.2	500	2000	0	70	60.2 ± 0.30
12	0.2	500	3000	0	70	60.4 ± 0.28
12	0.2	1500	1500	0	69	59.1 ± 0.32
12	0.2	3000	1500	0	69	59.3 ± 0.26
12	0.2	500	1500	45	71	61.4 ± 0.28
20	0.2	500	1500	0	68	58.4 ± 0.27

**Table 6:** ANOVA results of the arithmetic mean surface roughness Ra measured in the transverse direction.

Source	Sum of Squares	Degree of Freedom	Mean Square	F-Value	*p*-Value	Model Evaluation
Model	3.529716	3	1.176572	33.25087	2.28 × 10^−9^	significant
A—Step size	2.317558	1	2.317558	65.49605	8.22 × 10^−9^	
B—Feed rate	0.564782	1	0.564782	15.96119	0.000426	
E—side (inner, outer)	0.918013	1	0.918013	25.94377	2.15 × 10^−5^	
Residual	0.990772	28	0.035385			
Lack of fit	0.990772	22	0.045035			
Pure error	0	6	0			
Correlation total	4.520488	31				

**Table 7:** ANOVA results of the arithmetic mean surface roughness Ra measured in the longitudinal direction.

Source	Sum of Squares	Degree of Freedom	Mean Square	F-Value	*p*-Value	Model Evaluation
Model	5.63898	4	1.409745	21.08243	5.55 × 10^−8^	significant
A—Step size	3.825182	1	3.825182	57.20477	3.9 × 10^−8^	
B—Feed rate	0.745074	1	0.745074	11.14242	0.002471	
C—Tool rotational speed	0.340258	1	0.340258	5.088483	0.032393	
E—side (inner, outer)	1.220703	1	1.220703	18.25535	0.000214	
Residual	1.805442	27	0.066868			
Lack of fit	1.805442	21	0.085973			
Pure error	0	6	0			
Correlation total	7.444422	31				

## Data Availability

The original contributions presented in this study are included in the article. Further inquiries can be directed to the corresponding authors.
